# Blood neurofilament light chain and thrombospondin-1 levels of patients with autism spectrum disorder

**DOI:** 10.55730/1300-0144.5406

**Published:** 2022-05-07

**Authors:** Cem PAKETÇİ, Çağatay ERMİŞ, Ali Rıza ŞİŞMAN, Semra HIZ, Burak BAYKARA, Uluç YİŞ

**Affiliations:** 1 Department of Pediatric Neurology, Faculty of Medicine, Dokuz Eylül University, İzmir, Turkey; 2 Department of Child and Adolescent Psychiatry, Diyarbakır Children’s Hospital, Diyarbakır, Turkey; 3 Department of Medical Biochemistry, Faculty of Medicine, Dokuz Eylül University, İzmir, Turkey; 4 Department of Child and Adolescent Psychiatry, Faculty of Medicine, Dokuz Eylül University, İzmir, Turkey

**Keywords:** Neurofilament light chain, thrombospondin, autism, neurodegeneration

## Abstract

**Background/aim:**

Neurofilaments are intermediary filaments associated with neurodegenerative processes. Thrombospondin-1 (TSP-1) is a biological marker playing a role in synaptogenesis. This study aimed to investigate serum neurofilament light chain (NFL), and TSP1 levels of patients with autism spectrum disorder (ASD) compared to typically developing (TD) children.

**Materials and methods**: Forty-three patients with ASD and forty-five TD children were included. Serum biomarker levels were measured using the sandwich ELISA technique. The Childhood Autism Rating Scale (CARS) was implemented to measure the severity of ASD.

**Results:**

NFL and TSP1 levels did not differ between study groups (For NFL, ASD = 47.8 ± 11.4 vs. TD = 48.2 ± 15.3 pg/mL, p = 0.785; for TSP1, ASD = 224.4 ± 53.7 vs. TD = 224.7 ± 69.0 ng/mL, p = 0.828). Stereotyped behavior and sensory sensitivity domain of the CARS scale was negatively correlated with serum TSP-1 (r = −0.390, p = 0.010) and NFL (r = −0.377, p = 0.013) levels. Age was also positively correlated with NFL levels (r = 0.332, p = 0.030) in the ASD groups but not in the TD group.

**Conclusion:**

Our results did not support the neurodegenerative process of ASD. Future studies are needed to investigate neuroprogression in a longitudinal follow-up.

## 1. Introduction

Autism spectrum disorder (ASD) is a neurodevelopmental disorder characterized by deficits in social interaction, verbal and nonverbal communication, as well as restricted, repetitive patterns of behaviors and interests [1]. The underlying genetic etiology of ASD has been previously investigated, and many genes associated with neurogenesis, brain maturation, and synaptogenesis have been implicated in the pathophysiology of the disorder [2–4]. Neurodegenerative processes and immune system abnormalities have been proposed in the etiology of ASD [5,6]. Neuronal cell loss, microglia, and astrocyte activation increased proinflammatory cytokines, and oxidative stress might play a role in ASD, indicating the possible role of neurodegenerative processes [6,7]. Additionally, lymphocyte/monocyte ratio, platelet/lymphocyte ratio (PLR), and neutrophil/lymphocyte ratio (NLR) were examined as the peripheral biomarkers of inflammation, which could be rapidly obtained from routine blood tests [8,9]. Especially, NLR was proposed as a cost-effective measurement, which might have clinical implications [9]. However, these findings in the previous literature were limited. Despite being widely investigated, there is no established biomarker that reflects changes during the illness course [10]. ASD may involve some processes that occur in neurodegeneration; therefore, finding a biomarker remained a research endeavor to monitor clinical deterioration and prognosis.

Neurofilaments were structural elements of the cytoskeleton, composed of four subunits neurofilament light chain (NFL), neurofilament medium-chain (NF-M), neurofilament heavy chain (NF-H), and alpha-internexine or peripherin [11]. NFL was widely expressed in myelinated subcortical axons of the central nervous system (CNS) [12,13]. Previous studies demonstrated the structural and functional role of neurofilaments in synaptic junctions [13]. Also, neurofilaments were associated with axonal injury and traumatic brain damage [12–14].

In the previous literature, a recent study demonstrated that cerebrospinal fluid (CSF) and serum NFL could be a biomarker of neurodegeneration in mice [15]. Increased NFL in CSF was also associated with several neurologic conditions, including Alzheimer’s disease, amyotrophic lateral sclerosis, multiple sclerosis, inherited neuropathy, Parkinson’s disease, dementia, multiple system atrophy, and progressive supranuclear palsy, also predicting the prognosis of Alzheimer’s and Parkinson’s diseases [16–19]. Additionally, previous research revealed the association between sports-related repetitive head trauma and increased NFL within CSF, supporting the notion that the NFL might indicate chronic traumatic brain injury [14]. Neurofilaments could be reliably measured in CSF. Also, there was a significant association between serum and CSF neurofilament levels [20]. Accordingly, another study showed the correlation between plasma and CSF levels in patients with amyotrophic lateral sclerosis and blood NFL was nearly four times higher in the patient group than healthy controls, which also increases the risk of mortality as a prognostic biomarker [19]. In addition, recently, He and colleagues [21] revealed that serum NFL levels of patients with ASD were higher than typically developed (TD) children.

Thrombospondins (TSP) are matricellular regulator proteins that are dynamically expressed within the extracellular matrix (ECM) and implicated in the pathogenesis of several neurological illnesses [22]. TSP-1, initially considered a regulator protein of angiogenesis, plays several key roles in the angiogenic function, including apoptosis, cell-cell interaction, adhesion, and migration [22,23]. Besides, astrocytes were discovered to express TSP-1, which mediates synaptogenesis by interacting with neuroligin 1 in hippocampal neurons [22,24]. Given the synapse-modulating role of neuroligins, it has been proposed that there might be a possible link between secreted synapse-modulating glial proteins and impaired synaptic connectivity in ASD [25]. Furthermore, another study also suggested common variants of the *THBS1* gene, encoding thrombospondin 1 protein, could be protective against autism and rare variants pose a genetic predisposition to autism [26]. However, these results were not conclusive for the relationship between TSP-1 and autism since more studies are still needed to extend the clinical implications of previous findings.

Considering the increasing body of evidence for the use of NFL in neurologic and psychiatric conditions in the previous literature, we aimed to evaluate serum NFL levels of peripubertal patients with ASD compared to TD children. Likewise, given the potential role of TSP-1 in synaptogenesis, these novel biomarker candidates should be explored in ASD. To this end, we investigated serum NFL and TSP-1 levels in patients with ASD compared to typically developing children. We hypothesized children with ASD would yield higher levels of peripheral NFL and TSP-1 due to neuronal loss and synaptic disintegration. Second, we expected the severity and age were positively correlated with both peripheral biomarkers among the ASD group, indicating an inflammatory and neurodegenerative process in the course of autism. Finally, PLR and NLR were compared between patients with ASD and TD controls.

## 2. Method

### 2.1. Participants

Forty-three patients with ASD and 45 TD children were included in the study between May 2018–May 2020. Patients with ASD were registered and regularly followed children in the outpatient clinic of a tertiary-care university hospital. All clinical diagnoses were confirmed by an experienced clinician in neurodevelopmental disorders as per the Diagnostic and Statistical Manual of Mental Disorders-5 [1]. TD group, recruited from the pediatric outpatient clinic, consisted of healthy children who had similar age and sex characteristics. The healthy controls were examined for neurologic and psychiatric disorders. Subjects who had i) psychiatric disorders, ii) neurologic illnesses, and iii) any delay in developmental milestones were not included. Sociodemographic and illness characteristics were obtained from caregivers, and the severity of illness was measured using the Turkish version of the Childhood Autism Rating Scale (CARS) [27]. Inclusion criteria involved (i) being aged between 6–18 years and (ii) the total score of CARS ≥ 30 for the subjects with ASD. Patients with (i) genetic, neurodegenerative, inflammatory, autoimmune, and neoplastic diseases; (ii) the history of traumatic brain injury, (iii) the presence of ischemic brain illness, fever, renal or hepatic failure, recent or acute infection and (iv) the presence of mood disorders and psychotic disorders were excluded from the study. Informed consent was obtained from all parents and typically developing children. The patients were enrolled after obtaining written informed consent or assent (whenever applicable). The Local Ethical Committee of Dokuz Eylül University reviewed and approved the study protocol (number: 441-SBKAEK).

### 2.2. Procedures

Serum NFL and TSP-1 levels were measured with a commercial kit for sandwich Enzyme-Linked immune sorbent assay (For NFL, catalog number: YLA4279HU, assay range = 1.56–300 pg/mL, sensitivity = 0.54 pg/mL, CV: intra-assay < 10%, inter-assay < 12%; for TSP-1, catalog number: YLA1727HU, assay range = 5–700 ng/mL, sensitivity = 2.39 ng/mL, CV: intra-assay < 8%, inter-assay < 10%, Shangai YL Biotech Co.). Additional information was obtained from electronic patient files or treating physicians. Total blood count was also implemented. PLR and NLR parameters were calculated from total blood count results. Electroencephalography (EEG) at sleep states was recorded using scalp electrodes per International 10/20 system using a 16-channel EEG system for 30 min (EEG-9200K, Nihon Kohden Corporation, Tokyo). All records were evaluated by two pediatric neurology consultants independently. Findings were classified as (i) normal EEG, (ii) abnormal background activity, and (iii) epileptic abnormalities. When patients were compliant with procedures, Magnetic Resonance Imaging (MRI) at 1.5 T was implemented.

### 2.3. Statistical analysis

Power analysis (power = 0.90, alpha = 0.05) based on the previous research indicated 40 participants in each group [21]. The three-factor model of the CARS scale was used to measure the severity of (i) social communication, (ii) emotional reactivity, and (iii) stereotyped behavior and sensorial sensitivity subdomains of ASD [28].

Categorical variables were represented as frequencies and compared by using the chi-square test. Continuous variables were demonstrated as means and standard deviations. Normal distribution of continuous variables was controlled using skewness and kurtosis values. The independent-sample t-test was used to compare continuous demographic variables. Pearson correlation was also calculated to test the relationship between blood biomarkers and the characteristics of participants.

After subdividing participants into two different age categories (adolescents aged ≥ 12 years and children less than 12 years), we have implemented posthoc two-way ANOVA models using two categorical variables (i.e. diagnostic category and age category). The assumption for homogeneity of variances was tested using Levene’s test. In these two-way ANOVA models, diagnoses (i.e. ASD or TD controls) and age categories (i.e. children and adolescents) were variables to test their effect on serum NFL and TSP-1 levels.

Finally, posthoc subgroup analyses were conducted to compare patients receiving medications and those with MRI or EEG abnormalities by using the Mann-Whitney U test due to the sample size. Alpha was set at 0.05 two-tailed for statistical significance. All statistical analyses were performed in the SPSS software version 24.0 (IBM Corporation, Armonk, NY, USA).

## 3. Results

### 3.1. Demographic and illness characteristics

Table 1 demonstrates the sociodemographic and clinical characteristics of study participants. ASD and TD groups did not show any statistically significant difference for age and sex characteristics. In the ASD group, the mean age at the time of diagnosis was 3.3 ± 1.1 years. MRI scans (17.9%) and EEG examinations (24.3%) revealed abnormal findings among patients with autism. The total score of CARS was 41.7 ± 5.2 for the patient group.

### 3.2. Serum biomarker levels and blood count results of study participants

Blood count results and serum biomarker levels of study groups are shown in Table 2. The ASD group had a higher number of lymphocytes compared to the comparison group. The number of neutrophils and platelets was similar between the study groups. PLR was also significantly lower in the ASD group. Serum TSP-1 and NFL levels did not differ between study groups. In two-way ANOVA models including diagnosis (i.e. ASD or TD controls) and the age category (i.e. children or adolescents), there was also no significant effect of diagnosis (F = 0.1, p = 0.785), age category (F = 2.4, p = 0.124) or the interaction between both variables (F = 0.2, p = 0.625) on serum NFL levels. Likewise, diagnostic category (F = 0.0, p = 0.828), the age group (F = 2.3, p = 0.135) and the interaction effect (F = 0.0, p = 0.862) were not significant in the models to estimate serum TSP-1.

### 3.3. Correlation analysis between serum biomarkers, age, and the illness severity

The total CARS score was not statistically correlated with serum NFL and TSP levels (Table 3). The total score of stereotyped behavior and sensorial sensitivity was negatively correlated with serum NFL and TSP-1. Total social communication and emotional reactivity scores were not associated with NFL and TSP-1 levels. In the ASD group, age was positively correlated with serum NFL (r = 0.332, p = 0.030) and the correlation between age and TSP-1 was at trend level (r = 0.283, p = 0.066). In contrast, age did not show any significant correlation with NFL and TSP-1 in TD comparisons (For NFL, r = 0.003, p = 0.985; for TSP-1, r = 0.067, p = 0.664).

### 3.4. Subgroup analysis of patient receiving antipsychotic medications

In order to detect possible effects of medication on outcomes of interest, we implemented post-hoc subgroups analyses between patients receiving antipsychotic (n = 28) and antiepileptic (n = 7) medications among patients with ASD. Antipsychotic-user (65.1%) vs. nonuser (34.9%) groups did not differ regarding serum TSP-1 (antipsychotic-user group = 227.1 ± 57.0 vs. 219.4 ± 48.3 ng/mL, Z = 0.5, p = 0.637) and NFL (antipsychotic-user group = 47.7 ± 12.6 vs. 47.9 ± 9.2 pg/mL, Z = 0.8, p = 0.422). Similarly, patients receiving antiepileptic medication (16.3%) did not differ from nonuser group (83.7%) (For TSP-1, antiepileptic-user group = 227.1 ± 57.0 vs. 219.4 ± 48.3 ng/mL, Z = 0.5, p = 0.637; for NFL, antiepileptic-user group = 47.7 ± 12.6 vs. 47.9 ± 9.2 pg/mL, Z = 0.8, p = 0.422).

### 3.5. Subgroup analyses of patients with EEG and MRI abnormalities

Serum NFL levels did not differ between patients with normal MRI findings and those with abnormal MRI findings (patients with normal MRI findings = 44.5 ± 6.5 vs. patients with abnormal MRI = 46.6 ± 9.3 pg/mL, Z = 0.2, p = 0.834). Similarly, TSP-1 levels were similar between both subgroups (patients with normal MRI findings = 204.4 ± 32.1 vs. 225.1 ± 41.4 ng/mL, Z = 1.1, p = 0.280). In EEG assessments patient with abnormal background activity (n = 2) and epileptic abnormalities (n = 7) were grouped together to compare with those having normal EEG. Patients with normal EEG findings had higher levels of serum NFL compared to those with abnormal EEG findings (patients with normal EEG findings = 45.2 ± 8.8 vs. 49.3 ± 8.0 pg/mL, Z = 2.0, p = 0.045). Finally, TSP levels were similar in both patient subgroups (patients with normal EEG findings = 211.7 ± 43.5 vs. 233.3 ± 39.4 ng/mL, Z = 1.6, p = 0.111).

## 4. Discussion

Our study results suggested no difference between patients with ASD and typically developing children in terms of serum NFL and TSP-1 levels. Age was positively correlated with plasma NFL among patients with autism but not in controls. Stereotyped behavior and sensorial sensitivity scores were negatively correlated with serum NFL and TSP-1 levels. Conversely, the total CARS score, social communication, and emotional domain scores did not yield any correlation with both biomarkers. In posthoc subgroup analyses, antipsychotic and antiepileptic medications had no significant effect on TSP-1 and NFL. Finally, in the blood count analysis, lymphocytes and PLR were lower in ASD than in TD. To our knowledge, this is the first study that examines serum TSP-1 levels of patients with ASD in the literature.

Patients with ASD did not differ from the control group regarding plasma NFL as a biomarker of neural loss. This result was not in line with the previous study conducted by He and colleagues [21]. The mean age of the previous study was 5.1 years [21], which was lower than the mean age of our sample and might partially account for the discrepancy between the results given the significant correlation between age and serum NFL levels. Divergent findings of both studies could stem from demographic differences and ongoing medications. On the other hand, we did not confirm that the severity of autism was correlated with the serum NFL levels [21]. Yet, the age groups of both samples represent different neurodevelopmental stages of the brain. It might be argued that these discrepant results might be associated with varying pruning patterns in the brain. Accordingly, despite the modest sample size, the results of our study suggest that the neurodegenerative biomarker levels of ASD were similar to those of TD during the peripubertal period and early adolescence.

Loss of previously-acquired skills (also called “regression”) and/or structure of neurons were suggested as the signs of neurodegeneration, which might be related to increased proinflammatory cytokines, microglial activation, and oxidative stress leading to a type of progressive encephalopathy [6]. However, progressive encephalopathy might not be the case for relatively stable phases of ASD, or a slower rate of degeneration could remain undetectable in plasma. In line with our findings, a recent study did not find any difference between adult patients with major psychiatric disorders (e.g., schizophrenia, bipolar disorder, and major depressive disorder) and healthy comparisons [29]. Authors concluded that similar levels of NFL between psychiatric disorders and controls did not support the neurodegenerative process in the course of psychiatric disorders [29]. Likewise, another study involving children with progressive and nonprogressive neurological illnesses revealed NFL differentiated ongoing neural and/or axonal damage from stationary and stable neurologic diseases [30].

On the other hand, in the extant literature, children with autism were shown to have normal brain volume at birth, yielding an overgrowth in early childhood and total brain volume reached a plateau during adolescence within the range of typical development [31]. In addition, it was shown that patients with ASD had a sharper decline in total brain volume in late adolescence and young adulthood compared to typical development after the ages of 10–15 years [32]. Considering the mean age of our participants was ten years at the time of recruitment, chronologic age might be a confounding factor for the assessment of a neural biomarker. Besides, a preliminary study found a plasma glial fibrillary acidic protein levels in the ASD group were three times lower than those in the control group, and the mean age of the study group was approximately four years in that study [33]. Finally, we found a positive correlation between age and NFL in the patient group, but not in the TD children; therefore, it might be argued that this finding could be linked to the accelerated volumetric changes during adolescence among the ASD population.

Plasma TSP-1 was also found similar between study groups. As previously mentioned, TSP-1 was an essential protein of synaptogenesis and interacted with Neuroligin 1 during synapse formation [22, 24, 34]. Although common and rare variants of the *THBS1* gene were somewhat linked to ASD [26], it might be implicated in the early stages of brain development. Thus, despite its role in synaptogenesis, TSP-1 was not considered a peripheral biomarker of ASD. Additionally, TSP-1 has several regulatory and structural functions in the ECM, including angiogenesis, cell adhesion, and apoptosis [23]. Therefore, TSP-1 could be affected by pathological and physiological processes within the ECM of peripheral tissues. Accordingly, CSF levels of TSP-1 could indicate more conclusive results in younger patients.

Our study results did not suggest a robust relationship between biomarkers and the clinical characteristics of patients. We did not find any difference between patients receiving antipsychotic and antiepileptic medications. Antipsychotics had some anti-inflammatory effects on the immune system in rat models [35]. Of note, risperidone could cause immune alterations in the course of treatment [36]. Yet, the protective effects of existing medications from the neurodegenerative process are not solely concluded by these molecular findings, since cognitive impairment, the development of psychosis, or mood disorders could commonly occur during the transition to adulthood in the longitudinal follow-up [37].

There was a negative correlation between stereotypic behavior/sensorial sensitivity symptom scores and serum NFL and TSP-1. However, this relationship might be indirect since stereotypic behaviors and restricted interests were shown to be more prevalent in younger ages in the course of ASD, which seems a confounding factor [38]. Similarly, patients with EEG abnormalities had higher levels of NFL; yet, these findings were not controlled for age. Taken together, the connection between clinical characteristics and biomarkers requires further exploration.

The results of this study suggested white blood cell and lymphocyte counts were higher in patients with ASD compared to controls. In contrast to our results, a recent study proposed patients with ASD had elevated monocyte counts; nevertheless, lymphocytes and neutrophils were similar to those of controls, resulting in a lower lymphocyte/monocyte ratio in the patients with ASD [8]. However, the mean age of the case group in this study was 13.5 years, which could partially explain the discrepant results. Another study also suggested a higher neutrophil/lymphocyte ratio among the unmedicated patients with ASD than age- and sex-adjusted controls [9]. Given the inconsistency in the literature regarding blood cell counts, the diagnostic and prognostic values of blood cells are still unclear. Since the case groups of both studies did not receive any treatment, antipsychotics and antiepileptic medications used in our sample could alter the number of blood cells, leading to deviations from the previous findings.

Several limitations have to be taken into consideration to interpret the study results. First, the cross-sectional design of the study did not endorse repeated measures to reveal possible changes in serum levels. Yet, the mean age of the ASD group was 10.0 years, which allows us to find a response to the research question of whether neurodegeneration occurs during the peripubertal periods of patients with autism. Similarly, comorbidities and medication use might affect study results. However, posthoc tests suggested that medications did not have any significant effect on serum biomarker levels. On the other hand, we implemented correlation analyses to detect possible effects of the severity of symptoms, age, and medications. The diagnosis of ASD was not evaluated with a gold-standard instrument. However, a severity threshold of CARS scores ≥ 30 only endorsed moderate or severe cases to be included. Our sample size was also modest, especially for posthoc analyses. Therefore, some of the posthoc analyses were underpowered. Yet, we implemented a power analysis to determine the required sample size. Finally, blood levels might not reflect spinal fluid or the brain. Despite these limitations, to the best of our knowledge, this is the first study to compare serum TSP-1 levels between patients with ASD and TD children. Also, our results did not confirm elevated serum NFL levels in peripubertal patients with ASD. Considering the growing evidence in this field, the results of this research contributed to the current literature on biomarkers.

In conclusion, the NFL and TSP-1 did not differ between children with ASD and control groups. The results do not support the neurodegenerative process of patients with ASD during the peripubertal period. Designs with repeated neuropsychological and neuroimaging assessments, which could show the patterns of cognitive and functional decline, are needed to investigate the neurodegeneration of ASD.

## Figures and Tables

**Table 1:** Demographic and clinical characteristics of study participants.

Variables	ASD, n = 43	TD, n = 45	Statistics	*p*
Age, years, mean ± SD	10.0 ± 2.7	10.9 ± 3.2	*t* = 1.3	0.189
Sex, n (%)			χ^2^ = 1.2	0.277
Male	34 (79.1)	31 (68.9)		
Female	9 (20.9)	14 (31.1)		
Age at the time of diagnosis, years, mean ± SD	3.3 ± 1.1	-	-	*-*
Medications, n (%)				
Antipsychotic	28 (65.1)	-	-	*-*
Antiepileptic	7 (16.3)	-	-	*-*
MRI evaluation, n (%) a				
Normal	23 (82.1)	-	-	*-*
Abnormal findings b	5 (17.9)	-	-	*-*
EEG assessment, n (%) c				
Normal	28 (75.7)	-	-	*-*
Abnormal background activity	2 (5.4)	-	-	*-*
Epileptic abnormalities	7 (18.9)	-	-	*-*
CARS scores, mean ± SD				
Social communication	20.7 ± 2.7	-	-	*-*
Emotional reactivity	8.2 ± 1.4	-	-	*-*
Stereotyped behavior and sensory sensitivities	10.4 ± 1.7	-	-	*-*
Total CARS score d	41.7 ± 5.2	-	-	-

*Note* ASD = autism spectrum disorder, CARS = The Childhood Autism Rating Scale, EEG = electroencephalography, MRI = magnetic resonance imaging, SD = standard deviation, TD = typically developing children.

a This variable was available for 28 cases.

b Abnormal MRI findings involved periventricular leukomalacia (n = 2), cortical dysplasia (n = 1), and cerebral atrophy (n = 2).

c This variable was available for 37 cases.

d One item (visual response) did not load on any factor an was only counted in the total score.

**Table 2:** Biomarker levels and blood count results of study participants.

Variables	ASD	TD	Diagnosis		Age group		Interaction	
(mean ± SD)	n = 43 a	n = 45 a	*F*	*p*	*F*	*p*	*F*	*p*
WBC (10^3^/μL)			2.1	0.151	2.8	0.096	1.6	0.209
Children	8.2 ± 2.0	7.0 ± 1.5						
Adolescents	6.9 ± 2.2	6.8 ± 2.1						
Total	7.9 ± 2.1	6.9 ± 1.7						
Neutrophils (10^3^/μL)			0.3	0.614	0.5	0.500	0.4	0.516
Children	4.1 ± 1.5	3.7 ± 1.4						
Adolescents	3.7 ± 1.5	3.7 ± 1.4						
Total	4.0 ± 1.5	3.7 ± 1.4						
Lymphocytes (10^3^/μL)			5.0	**0.027**	6.6	**0.012**	3.1	0.082
Children	3.2 ± 0.9	2.5 ± 0.6						
Adolescents	2.4 ± 0.7	2.3 ± 0.7						
Total	3.0 ± 0.9	2.4 ± 0.6						
Platelets (10^3^/μL)			2.7	0.105	2.5	0.115	1.0	0.310
Children	322.6 ± 84.6	333.8 ± 55.0						
Adolescents	275.6 ± 82.6	323.5 ± 71.0						
Total	312.8 ± 85.4	330.6 ± 59.8						
NLR			0.6	0.439	0.1	0.708	0.2	0.636
Children	1.4 ± 0.7	1.7 ± 1.2						
Adolescents	1.6 ± 0.6	1.6 ± 0.5						
Total	1.4 ± 0.7	1.6 ± 1.0						
PLR			6.6	**0.012**	1.3	0.250	0.9	0.345
Children	105.5 ± 33.1	142.5 ± 42.3						
Adolescents	127.7 ± 76.6	144.7 ± 35.0						
Total	110.1 ± 45.4	143.2 ± 39.8						
TSP1 (ng/mL)			0.0	0.828	2.3	0.135	0.0	0.862
Children	219.0 ± 45.2	218.3 ± 67.6						
Adolescents	244.8 ± 78.3	238.8 ± 72.4						
Total	224.4 ± 53.7	224.7 ± 69.0						
NFL (pg/mL)			0.1	0.785	2.4	0.124	0.2	0.625
Children	46.4 ± 9.6	47.1 ± 13.8						
Adolescents	53.2 ± 16.2	50.6 ± 18.6						
Total	47.8 ± 11.4	48.2 ± 15.3						

*Note* Data was presented as mean ± standard deviations. ASD = autism spectrum disorder, NFL = Neurofilament light chain, NLR = Neutrophil/lymphocyte ratio, PLR = Platelet/lymphocyte ratio, TD = typically developing children, TSP1 = Thrombospondin 1, WBC = White blood cell.

a The ASD group is consisted of 34 children and 9 adolescents and the TD group includes 31 children and 14 adolescents.

**Table 3:** Correlation between blood biomarkers and the severity of illness among 43 children with ASD.

	Social communication score		Emotional reactivity score		Stereotyped behavior and sensorial sensitivity score		Total CARS score	
Variables	*Corr. coeff*.	*p*	*Corr. coeff*.	*p*	*Corr. coeff*.	*p*	*Corr. coeff*.	*p*
NFL (pg/mL)	−0.100	0.525	0.008	0.961	−0.377	**0.013**	−0.214	0.169
TSP-1 (ng/mL)	−0.080	0.609	0.070	0.657	−0.390	**0.010**	−0.191	0.219

*Note* CARS = The Childhood Autism Rating Scale, *Corr. Coeff = Correlation coefficient*, NFL = Neurofilament light chain, TSP-1 = Thrombospondin-1
